# Factors and costs associated with removal of a newly established population of invasive wild pigs in Northern U.S.

**DOI:** 10.1038/s41598-020-68264-z

**Published:** 2020-07-13

**Authors:** Justin W. Fischer, Nathan P. Snow, Bradley E. Wilson, Scott F. Beckerman, Christopher N. Jacques, Eric H. VanNatta, Shannon L. Kay, Kurt C. VerCauteren

**Affiliations:** 1 United States Department of Agriculture, Animal and Plant Health Inspection Service, Wildlife Services, National Wildlife Research Center, 4101 LaPorte Avenue, Fort Collins, CO 80521 USA; 2 United States Department of Agriculture, Animal and Plant Health Inspection Service, Wildlife Services, 3430 Constitution Drive, Suite 121, Springfield, IL 62711 USA; 3 Western Illinois University, 1 University Circle, 338 Waggoner Hall, Macomb, IL 61455 USA; 4 University of Wyoming, 1000 E University Ave, Laramie, WY 82071 USA; 5 Department of Statistics, Colorado State University, Fort Collins, CO 80521 USA

**Keywords:** Invasive species, Behavioural ecology, Animal behaviour

## Abstract

The human-mediated spread of exotic and invasive species often leads to unintentional and harmful consequences. Invasive wild pigs (*Sus scrofa*) are one such species that have been repeatedly translocated throughout the United States and cause extensive damage to natural ecosystems, threatened and endangered species, agricultural resources, and private lands. In 2005, a newly established population of wild pigs was confirmed in Fulton County, Illinois, U.S. In 2011, a state-wide wild pig damage management program involving federal, state, and local government authorities directed a concerted effort to remove wild pigs from the county until the last wild pig (of 376 total) was successfully removed in 2016. We examined surveillance data from camera traps at bait sites and records of wild pig removals during this elimination program to identify environmental and anthropogenic factors that optimized removal of this population. Our results revealed that wild pigs used bait sites most during evening and nocturnal periods and on days with lower daily maximum temperatures. Increased removals of wild pigs coincided with periods of cold weather. We also identified that fidelity and time spent at bait sites by wild pigs was not influenced by increasing removals of wild pigs. Finally, the costs to remove wild pigs averaged $50 per wild pig (6.8 effort hours per wild pig) for removing the first 99% of the animals. Cost for removing the last 1% increased 84-fold, and averaged 122.8 effort hours per wild pig removed. Our results demonstrated that increased effort in removing wild pigs using bait sites should be focused during periods of environmental stress to maximize removal efficiency. These results inform elimination programs attempting to remove newly established populations of wild pigs, and ultimately prevent population and geographic expansion.

## Introduction

There has been a dramatic surge in invasive wild pig (*Sus scrofa*) populations, geographic range, and ecological and economic impact in the United States over the last three decades^1–4^. Wild pigs cause millions of dollars in damage annually^5–7^, negatively affecting native habitats, endangered species, agricultural crop and livestock production, and transmitting diseases^1,8,9^. To counter these negative impacts, state (e.g., Illinois Department of Natural Resources, Missouri Department of Conservation) and federal (U.S. Department of Agriculture, Animal and Plant Health Inspection Service (USDA-APHIS)) resource management agencies have implemented wild pig damage management programs^10–12^. The primary goal of these programs is to minimize ecological and economical damage inflicted by wild pigs, which often necessitates lethal removal.

Wild pigs are extremely adaptable, having the ability to flourish in many environmental and anthropogenic regimes. Wild pigs can reach sexual maturity at a young age, have high annual growth rates, and have very few natural predators, all of which contribute to population growth and expansion^13–15^. Environmental predictors associated with the current distribution and potential future spread of wild pigs have been well documented^3,4,16^. Anthropogenic drivers of the current range of wild pigs include crop production^17–19^, habitat alteration^1,20,21^, hunting^22–24^, and the deliberate and illegal transport and release of wild pigs^1,25,26^. Climatic factors, landscape structure, desire for hunting opportunities, and public perception of the risks associated with wild pigs all play a role in their distribution.

Wild pig elimination programs are currently being implemented across much of the U.S. as part of the USDA-APHIS-Wildlife Services-National Feral Swine Damage Management Program^27^. Various removal strategies (i.e., hunting, trapping, and professional shooting) have been used to successfully eradicate wild pigs from limited areas^28–30^. Camera traps are a primary surveillance tool used to identify where wild pigs are located^31–33^ and determine optimal removal techniques (e.g., trap type, ground or aerial shooting, combinations thereof). Once the removal of wild pigs has been completed, a monitoring program for detection of any remaining or new wild pigs is vital to ensuring and maintaining eradication^29^.

The success of removal programs often hinges upon how removal techniques alter behavior and detection of wild pigs, especially at low densities^34,35^. Our objectives were to identify factors that contributed to the successful elimination of a newly established population of wild pigs during January 2012–January 2016 in Fulton County, Illinois, U.S. by: (1) determining which environmental and anthropogenic factors influenced daily use of bait sites, (2) determining which factors influenced monthly removal success, and (3) evaluating the costs associated with removal of the population throughout the elimination program. We considered this an exploratory case study because we evaluated removal data after all removals were completed. Throughout the entire elimination program, federal and state officials removed 376 wild pigs by trapping, ground shooting, and aerial shooting, being persistent until the last known wild pigs were removed from Fulton County^12^.

## Materials and methods

### Study area

**Figure 1:**
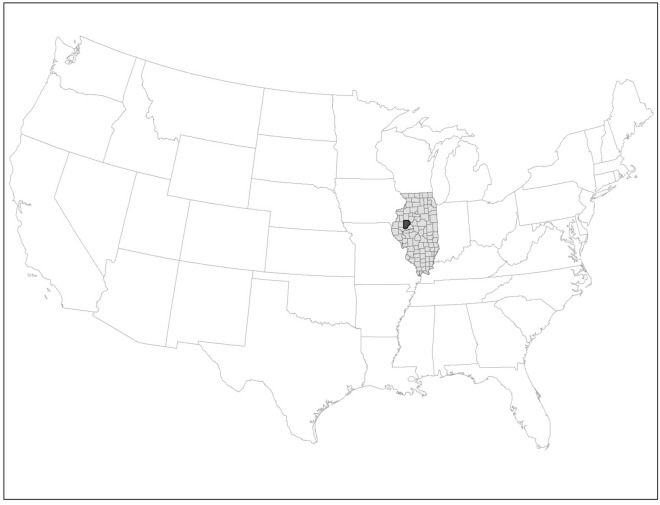
Fulton County, west-central Illinois, U.S.

Our study area occurred in Fulton County, west central Illinois, U.S., and was approximately 59 km^2^ in size (Fig. 1). The area was all private property and comprised of a rural landscape with a highly fragmented mix of forested and agricultural lands. First reports of wild pigs in Illinois occurred in the early 1990s in several southern counties and were the result of escaped livestock or released pets. Confirmed sightings of wild pigs in this county ensued in 2005 and it was believed one localized population of wild pigs inhabited Fulton County.

Annual mean monthly temperature was 11 °C with a mean January low temperature of − 18 °C and a mean July high temperature of 36° C (National Centers for Environmental Information; https://www.ncdc.noaa.gov/). Mean annual precipitation was 97 cm (National Centers for Environmental Information; https://www.ncdc.noaa.gov/).

### Camera trapping and removal of wild pigs

By 2011, an aggressive joint wild pig elimination program was initiated between the Illinois Department of Natural Resources (IDNR) and USDA-APHIS-Wildlife Services. We compiled camera trap data collected by the USDA-APHIS-Wildlife Services elimination program during January 2012–April 2014. To monitor for confirmed presence of wild pigs, one remote camera (Bushnell Trophy Cam (Bushnell Outdoor Products, Overland Park, KA), HCO Scoutguard (HCO Outdoor Products, Norcross, GA), Moultrie (PRADCO Outdoor Brands, Calera, AL), or Reconyx (Reconyx Inc., Holmen, WI)) was deployed at each site with signs of recent wild pig activity (i.e., rooting, rubs, wallow, or concentrated tracks) or areas where pig activity had been reported. Depending on camera availability and the number of sites with documented wild pig activity, between 1–20 sites were monitored concurrently. The remote cameras were programmed to record motion-activated images; they were programmed with multiple delay settings between pictures (i.e., 4–60 s) and numbers of pictures taken per event (i.e., 1–3) because of differences in manufacturer settings amongst the cameras. Cameras were typically mounted on trees or t-posts 90 cm above ground level overlooking a bait pile of soured corn. Initial bait amounts consisted of 38 L and sites were rebaited twice a week or as needed with approximately 8–15 L per visit.

The IDNR and Wildlife Services used images from cameras to assist with the wild pig elimination program. Once wild pigs were detected at a bait site, the IDNR and Wildlife Services attempted to remove them. Removal strategies primarily consisted of trapping and ground shooting, and to a lesser degree aerial shooting. If wild pig visitation to a bait site resulted in consistent daily activity, trapping or ground shooting removal techniques were employed based on group size and potential trap shyness. Ground shooting was often conducted at night with thermal or night vision equipment when only 1–2 wild pigs were observed visiting a bait site. Trapping was conducted using corral traps and when > 2 wild pigs were observed visiting bait sites. Corral traps were gradually erected over multiple days (i.e., 5–10 days) to provide time for wild pigs to acclimate to their presence. Traps were set after all visiting wild pigs became conditioned to them. Aerial shooting only occurred in February and March 2014. By this time the remaining wild pigs were difficult to locate and attract to bait sites; aerial shooting was believed to be the most efficient removal technique for this situation^12^. The IDNR and Wildlife Services regularly communicated with landowners for access to private properties, and estimated that landowners removed very few (i.e., < 5) wild pigs throughout the entire elimination program, thus landowner removals were not included in further analysis. Procedures for the elimination program were carried out in accordance with approved guidelines and regulations, and approved by a 2016 USDA-APHIS-Wildlife Services Environmental Assessment (Mammal Damage Management in the State of Illinois).

All images included a unique ID, date, and time stamp and were examined for presence of wild pigs. We organized images in a database and recorded the date and time of each visit by wild pigs to a given bait site and the number of wild pigs per image. All images were grouped into series of feeding bouts, where independent bouts were separated by ≥ 60 min of no visitation by wild pigs^36,37^. We calculated the length of each feeding bout (min) as the difference between the start and finish time for a series of consecutive images. We defined this time period (i.e., feeding bout) as a measure of duration of time that wild pigs spent at bait sites. We also identified the maximum number of wild pigs observed within a single image during each feeding bout at each site, to examine for influences from the numbers of wild pigs using bait sites through time. We considered this index an unbiased method for detecting changes in abundance at bait sites through time, though it likely underestimated the true abundance.

### Factors influencing daily use of bait sites

We considered the length of feeding bouts (i.e., response variable) as representing a metric of the use of bait sites by wild pigs throughout the camera monitoring period. The minimum duration we considered was zero min, reflecting wild pigs observed on camera but passing by a bait site. We used these durations of visitation to examine how time of day (i.e., based on when the feeding bout began (0001–2400 h)), daily climatic variables (described below), and seasonal predictors (described below) influenced the duration of visits by wild pigs at bait sites. Wild pigs have been reported to have increased movements and activity levels during nocturnal periods^22,23^ and activity patterns may be affected by temperature, precipitation, and relative humity^16,19^. We also included a quadratic term for time of day to account for a hypothesized non-linear relationship with feeding bout visitations.

For climatic predictors, we compiled daily climatic variables from the Global Historical Climatology Network (NOAA National Climatic Data Center; https://doi.org/10.7289/V5D21VHZ), including daily averages of precipitation (mm) and minimum and maximum temperatures (°C) so we could determine how these predictors influenced duration of visitations. For seasonal predictors, we defined two phenological seasons based on the growth stages of corn, the dominant crop type in the study area and an important food and shelter resource for wild pigs^17,38^. Seasons were considered as either: corn forage season (July 7–Nov 5) or non-corn forage season (Nov 6–July 6). Though it is recognized wild pigs consume corn shortly after planting^39^, our corn forage season reflects when ears are forming and the fields also serve as cover. The mean corn silking date in Fulton County was 7 July (Useful to Usable (U2U): Transforming Climate Variability and Change Information for Cereal Crop Producers; https://mygeohub.org/groups/u2u), and represented a critical stage in corn development when wild pigs begin to forage on ears of corn^38^. The mean corn harvest date was 5 November, by which time ~ 85% of corn is harvested in Illinois^40^ and represented the annual removal of corn as a food and cover resource for wild pigs.

We scaled and centered all numeric predictors to evaluate which of these variables had the largest effect on the duration of time wild pigs spent at bait sites. We conducted an intercorrelation analysis to exclude any predictor(s) in any correlated pair (i.e., |r|> 0.50) in Program R^41^. We excluded the daily minimum temperature and seasonal predictors because these variables were correlated with daily maximum temperature. We fit a Tweedie generalized linear mixed model using the cplm^42^ and lme4^43^ packages in Program R^41^. We examined all combinations of models stemming from the global model of:

$$time \, \,spent \, \,at\, \, a\, \, bait\, \, site\,\sim \,time \, \,of \, \,day\, + \,time \, \,of\, \, day^{2} \, + \,max\, \, temperature\, + \,precipitation.$$

We considered bait site ID and day as random effects in these models to account for repeated observations taken from the same sites through time.

We used the MuMIn package^44^ in Program R^41^ to calculate Akaike’s Information Criterion adjusted for small sample sizes (AIC_c_) to rank the 16 models in the model set (including a null model). We selected all models with ΔAIC_c_ ≤ 2.0 as having support for being the highest-ranked model^45^ for predicting the duration that wild pigs spent at bait sites. From the highest-ranked models, we evaluated relative importance of each variable within the model set, and considered any variable with relative importance < 30% as having weak support for influencing the duration that wild pigs spent at bait site^45^. For the highest-ranked models, we model averaged and examined the 95% confidence intervals (CI) surrounding the conditional regression coefficients (*β*) for lack of overlap with zero to provide statistical evidence for which predictors influenced usage of bait sites by wild pigs. We used conditional coefficients (i.e., the zero method^46^) because our aim was to determine which predictors had the strongest effect on the duration that wild pigs spent at bait sites^47,48^. We also implemented a paired bootstrapping algorithm to conduct 500 bootstrapped simulations to generate predicted effects and 95% predicted CIs for each variable, holding all other predictors constant at their means.

### Factors influencing monthly removals of wild pigs

We conducted an analysis of how monthly climate, elimination program effort, and number of wild pigs observed influenced the success of monthly pig removals. We used the temporal scale of months (i.e., 27 months (January 2012–April 2014) because this was the scale at which Wildlife Services removal effort data was compiled. For climatic predictors, we compiled daily averages of temperature and precipitation described above into monthly averages. For amount of effort, we used total number of hours reported by the elimination program spent afield during 2012–2014 removing wild pigs. These hours included time spent ground shooting, setting and checking bait sites, setting and checking traps, aerial shooting, and any reconnaissance locating wild pigs.

In addition to the hours of effort removing wild pigs, we also explored whether the number of hours reported by the elimination program conducting outreach to the public about the program may have influenced monthly removals. Outreach hours included time spent at public community events, landowner meetings, and radio and television interviews by elimination program employees. For example, employees presented information highlighting the destructive nature and negative consequences of wild pigs, as well as provided information on identifying presence of wild pigs, their legal status, reporting procedures, and where to get further information. The goal of the outreach was to identify locations of new groups of wild pigs and then attempt to remove those animals. We used a lag period of 1 month for the outreach hours under the hypothesis that monthly removal of wild pigs during a month (*t*) would be influenced by the number of outreach hours spent during the prior month (*t *− 1). Lastly, for the number of wild pigs observed, we extracted data from camera imagery representing the maximum number of wild pigs observed during a single feeding bout each month. This maximum represented the largest group size of wild pigs that were targeted for removal each month.

We conducted an intercorrelation analysis of all predictors as described above, and no predictors were required to be removed from the analysis. We used base package of Program R^41^ to explore all combinations of linear models stemming from the global negative binomial model of:

$$\begin{gathered} monthly\,count\,wild\,pigs\,removed\,\sim \,average\,temperature\, + \,average\,precipitation\, + \,hours\,of \hfill \\ removal\,effort\, + \,lag\,hours\,of\,outreach\, + \,maximum\,number\,of\,wild\,pigs\,per\,bait\,site. \hfill \\ \end{gathered}$$

We evaluated and ranked the subsequent 32 models in the model set (including a null model) similar to methods described above. We used the predict.lm function in the base package of Program R^41^ to generate predicted effects and 95% predicted CIs for each variable in the top-ranked model, holding all other predictors constant at their means.

We conducted two final analyses post hoc to examine how continual removal of the population of wild pigs influenced their use of bait sites through time. We hypothesized that wild pigs may have become more wary of bait sites and used them more sparingly or more erratically as more of the population was removed. Specifically, we used a linear model to examine how the cumulative total of wild pigs removed influenced the average amount of time (min) that wild pigs spent at bait sites each month. Secondly, we used a similar model to examine how the cumulative total of wild pigs removed influenced the variation (variance) of time that wild pigs spent at bait sites each month.

### Economics of removing wild pigs

We estimated the economic costs of removing wild pigs by multiplying the monthly effort hours described above by the average total cost per WS employee (i.e., $USD 31.33 per hour adjusted for 2019 inflation^49^). We similarly estimated the economic cost of aerial shooting removal efforts ($USD 626.58 per hour adjusted for 2019 inflation^50^), which included pilot and gunner salaries and helicopter operational cost. We used a gamma distributed generalized linear model with a log-link to examine how the costs of removing wild pigs were influenced by the percent of the population of wild pigs that were removed each month. We used the predict function to generate predicted effects on costs and 95% CIs.

## Results

We obtained 296,933 images from 708 bait nights at 90 unique bait sites with wild pig visitations during January 2012–April 2014. No images were collected during February 2014 but baiting and removal activity did occur, thus we excluded this month from our daily and monthly analysis.

### Factors influencing daily use of bait sites

**Figure 2:**
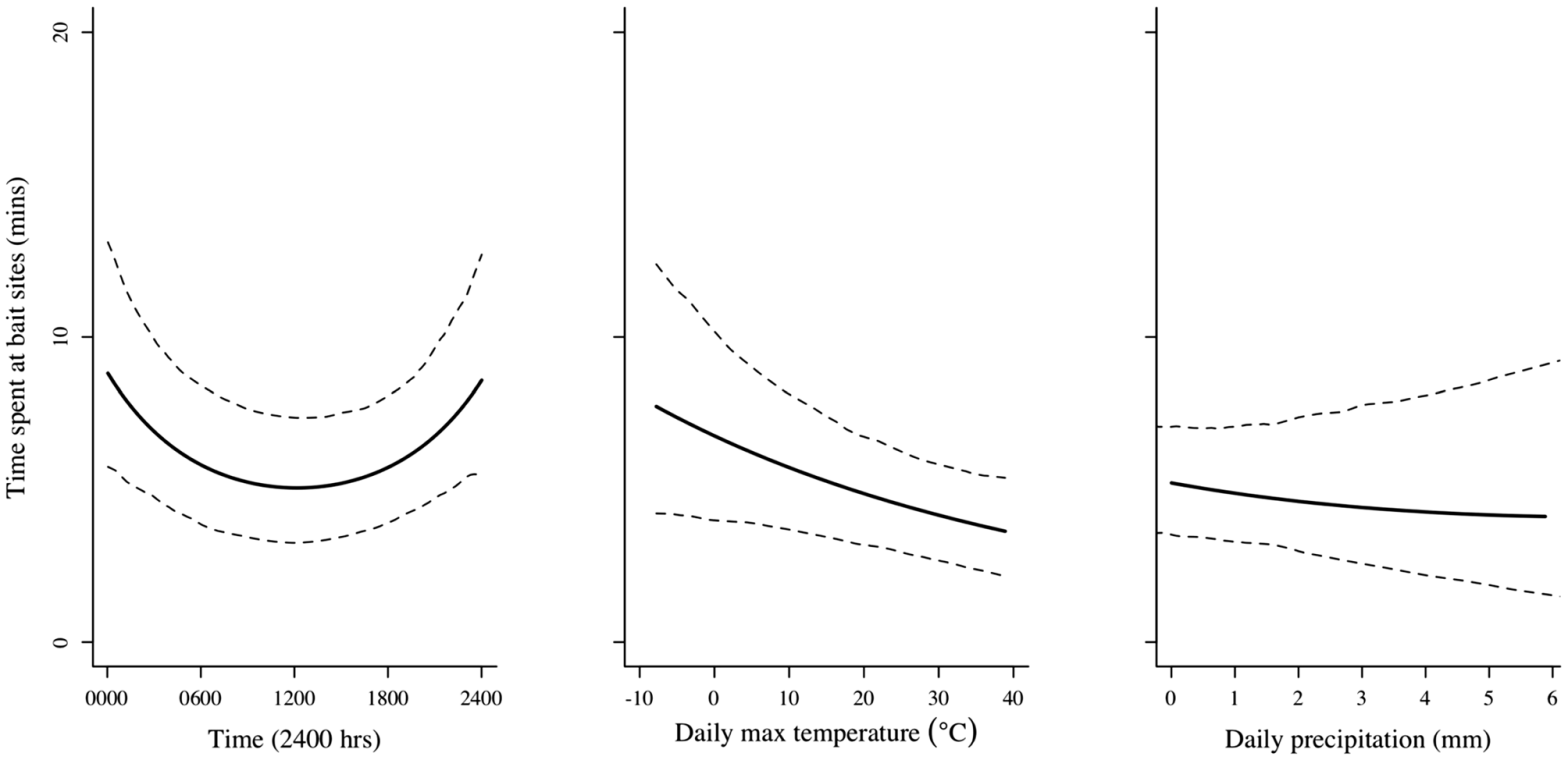
Effect plots from predictors in top-ranked model for describing the amount of time that wild pigs spent at bait sites during an elimination program in Fulton County, Illinois, U.S. during January 2012–April 2014.

Wild pigs spent an average of 33.19 min (SD = 51.4) during a feeding bout at bait sites, though visits ranged from 0–447 min. The model selection procedure indicated uncertainty for selecting the highest-ranked model for predicting the amount of time that wild pigs spent at bait sites (Table 1). Model averaging indicated that time of day, time of day^2^, precipitation, and maximum daily temperature were the most relatively important predictors (Table 2). Specifically, wild pigs used bait sites longer during evening and nocturnal periods, and when maximum daily temperatures and daily precipitation were lower (Table 2, Fig. 2).

**Table 1:** Highest-ranked linear mixed models for predicting the amount of time that wild pigs spent at bait sites during an elimination program in Fulton County, Illinois, U.S. during January 2012–April 2014.

Model^a^	K^b^	AIC_c_^c^	ΔAIC_c_^d^	*w*_*i*_^e^
Duration ~ Time of day + Time of day^2^ + Max temp	7	8,652.0	0.00	0.529
Duration ~ Time of day + Time of day^2^ + Max temp + Precip	8	8,653.9	1.94	0.201

^a^Time of day = time of day that feeding bouts started, Max temp = daily maximum temperature (degrees C), Precip = daily sum of precipitation (mm).

^b^No. of parameters.

^c^Akaike’s Information Criterion adjusted for small sample sizes (Burnham and Anderson 2002).

^d^Difference in AIC_c_ relative to minimum AIC_c_.

^e^Akaike weight (Burnham and Anderson 2002).

**Table 2:** Parameter estimates, uncertainty, and relative importance for highest-ranked predictors for describing the amount of time that wild pigs used bait sites, and the monthly count of wild pigs removed during an elimination program in Fulton County, Illinois, U.S. during January 2012–April 2014.

Parameter	Estimate	Conditional SE	95% CI		Relative importance
			Lower	Upper	
Time spent at bait sites					
Time of day	0.030	0.045	− 0.058	0.118	1.00
Time of day^2^	0.279	0.091	0.102	0.457	1.00
Max temp	− 0.211	0.083	− 0.373	− 0.050	1.00
Precip	− 0.016	0.048	− 0.109	0.078	0.28
Monthly removal of wild pigs					
Average temp	− 0.329	0.142	− 0.623	− 0.035	0.68
Max pigs	0.785	0.438	− 0.121	1.690	0.51
Effort	0.112	0.053	0.002	0.222	0.45

### Factors influencing monthly removals of wild pigs

**Figure 3:**
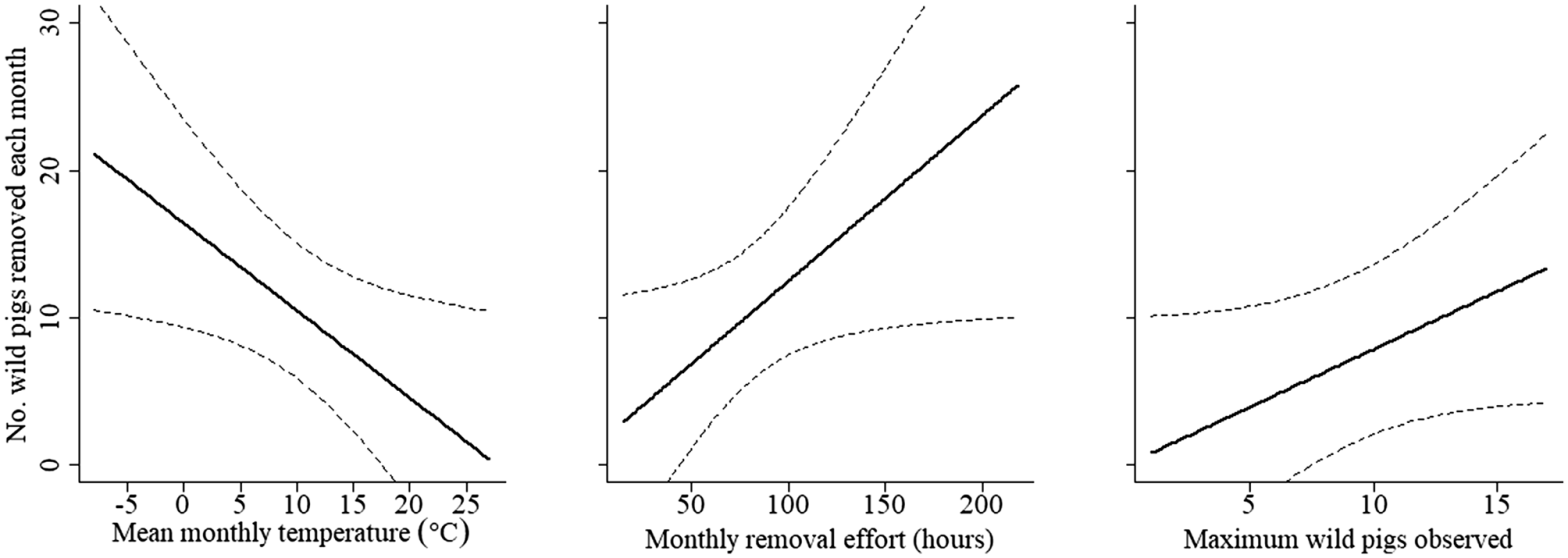
Effect plots from predictors in top-ranked model for describing monthly count of wild pigs removed during an elimination program in Fulton County, Illinois, U.S. during January 2012–April 2014.

A total of 313 wild pigs (83% of the total removed during the elimination program) were removed during January 2012–April 2014; 192 with trapping, 82 with ground shooting, and 39 with aerial shooting. On average, 10.4 wild pigs (SE = 2.5) were removed each month, but ranged from 0–51. This model selection procedure also indicated uncertainty for selecting a highest-ranked model. Model averaging indicated that average monthly temperature, amount of effort hours spent trying to remove wild pigs, and maximum number of wild pigs observed at bait sites each month were important predictors of monthly pig removal (Table 3). Specifically, more wild pigs were removed during months with lower average temperatures, and when more hours were spent trying to remove them (Table 2, Fig. 3). The maximum number of wild pigs observed did not statistically influence the count of wild pigs that were removed, but trended toward a positive relationship (*t*_23_ = 1.792, *p* = 0.086).

**Table 3:** Highest-ranked linear models for describing monthly count of wild pigs removed during an elimination program in Fulton County, Illinois, U.S. during January 2012–April 2014.

Model^a^	K^b^	AIC_c_^c^	ΔAIC_c_^d^	*w*_*i*_^e^
Removed ~ Temp + Effort + Max pigs	4	217.21	0.00	0.14
Removed ~ Temp + Effort	3	217.70	0.49	0.11
Removed ~ (.)	1	217.98	0.77	0.09
Removed ~ Max pigs	2	218.18	0.97	0.08
Removed ~ Temp	2	218.61	1.40	0.07
Removed ~ Temp + Max pigs	3	218.96	1.76	0.06

^a^Temp = mean monthly temperature, Effort = cumulative monthly hours spent removing wild pigs, Max pigs = mean monthly maximum number of wild pigs observed from camera imagery during a feeding bout.

^b^No. of parameters.

^c^Akaike’s Information Criterion adjusted for small sample sizes (Burnham and Anderson 2002).

^d^Difference in AIC_c_ relative to minimum AIC_c_.

^e^Akaike weight (Burnham and Anderson 2002).

**Figure 4:**
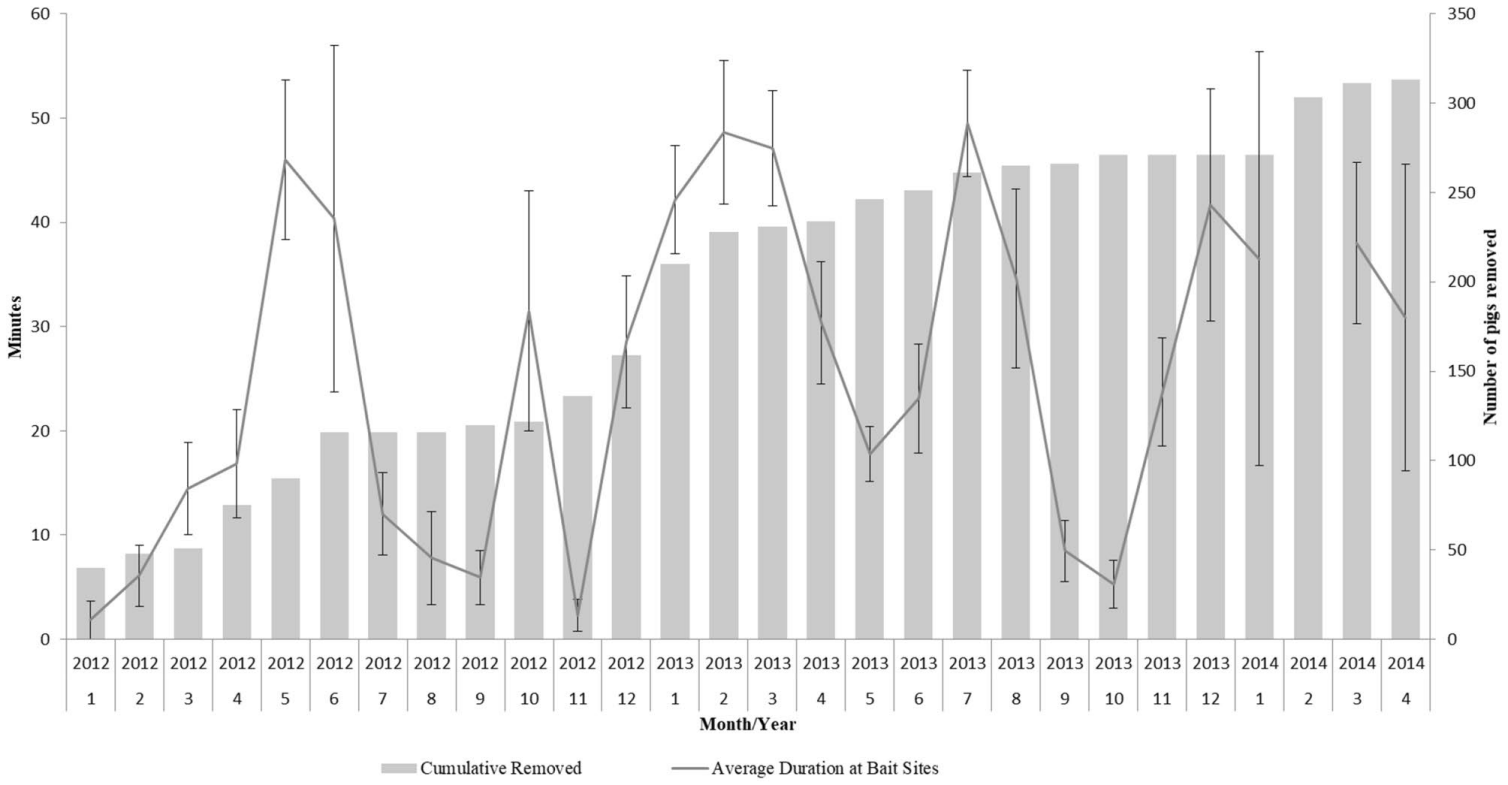
Average duration of time (± SE) wild pigs spent at bait sites and the cumulative number of wild pigs removed per month during an elimination program in Fulton County, Illinois, U.S. during January 2012–April 2014.

Lastly, we found no evidence that the continual removal of the population of wild pigs influenced their behaviors at bait sites through time. Specifically, as cumulative removals increased, we found no differences in the average time that wild pigs spent at bait sites (β = 0.01; 95% CI = − 0.01 to 0.04), or variation in their time spent at bait sites (β = 3.46; 95% CI = − 0.13 to 7.06; Fig. 4).

### Economics of removing wild pigs

**Figure 5:**
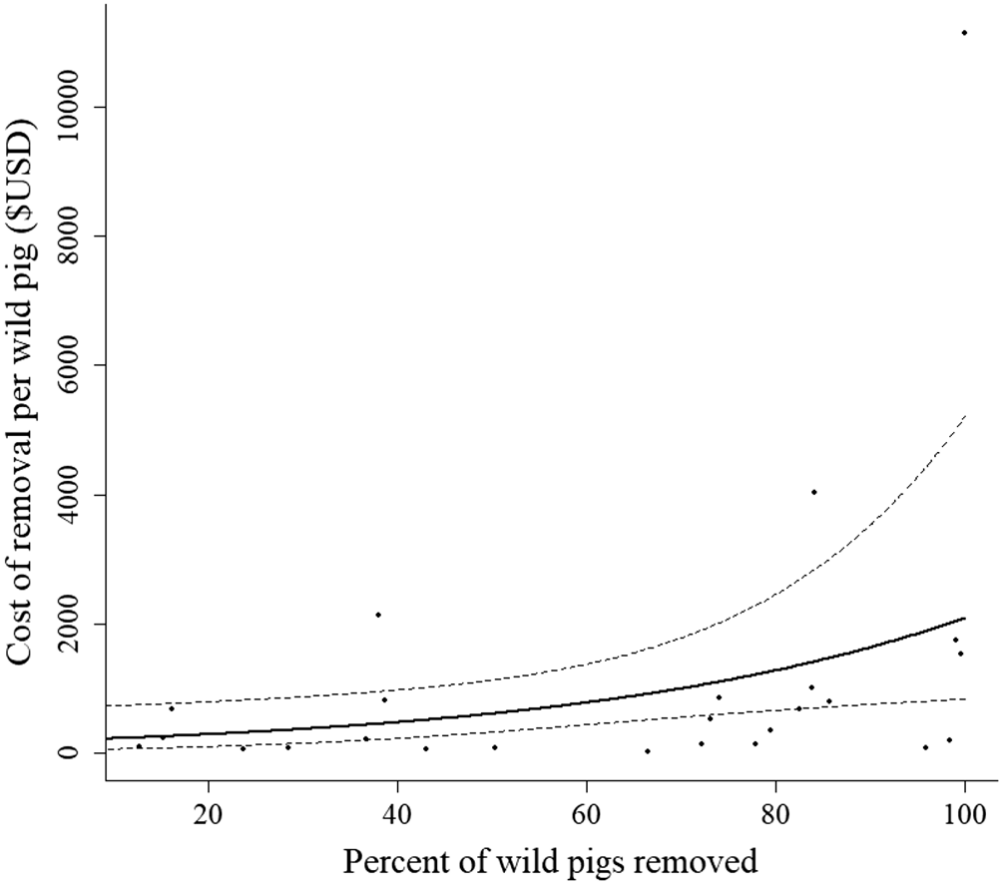
The predicted cost ($USD/wild pig) and 95% confidence intervals (dashed lines) for removing wild pigs during an elimination program in Fulton County, Illinois, U.S. during January 2012–January 2016. Points represent raw data used to fit the model.

In addition to the 313 wild pigs removed during January 2012–April 2014, the final three wild pigs were not removed until May 2014 (n = 2) and January 2016 (n = 1), respectively, despite continued effort. Not surprisingly, costs of removing the last remaining pigs increased as more of the population was removed (β = 0.02; 95% CI = 0.006–0.04; Fig. 5). The model indicated that for every one percent increase in the population removed, the costs of removal increased by two percent. However, the raw data showed the costs to remove wild pigs averaged $50 per wild pig (6.8 effort hours per wild pig) for removing the first 99% of the animals. Cost for removing the last 1% increased 84-fold, and averaged 122.8 effort hours per wild pig removed.

## Discussion

Wild pigs used bait sites longer during periods of lower temperatures indicating that alternative forage resources may be limited (i.e. winter). This suggests that elimination programs should focus their use of bait sites during time periods when wild pigs might be experiencing thermal or food stress, to most effectively remove them. In particular, the population of wild pigs in Fulton County, Illinois was among the northernmost populations of wild pigs in the U.S., thus was susceptible to colder winter temperatures and shorter growing seasons than most wild pigs in the U.S. In support of this, we found that removals of wild pigs were highest during periods of colder temperatures, likely because bait sites were more effective at attracting wild pigs.

Increased use of bait sites during periods of climatic stress is supported by other studies where wild pigs and boars alter their behaviors during times of year when caloric or habitat requirements are not being met. Wild boar in Spain seek urban areas for anthropogenic food resources during warmer, drier months^51–53^. High daily temperatures have been reported to limit movements of wild pigs in North America^54,55^ and Australia^56^, which could limit foraging efficiency^57^. Additionally, Snow et al*.*^4^ found that wild pigs were less likely to spread into regions with colder winters in the U.S. In Europe, seasonal drying of wetlands and marsh areas resulted in wild boar moving into cropland areas for food and wallowing sites^58^.

We also found that wild pigs used bait sites more during evening and nocturnal periods, which coincides with nocturnal behaviors, particularly for persecuted animals. Efforts to remove wild pigs have resulted in modification of behaviors to avoid detection by becoming more nocturnal^59^ and increasing movement rates^22–24^. Snow et al*.*^60^ reported that wild pig visitation rates to bait sites for a hunted population in south-central Texas peaked between 1900–0200 h. However, shifts in behaviors have not been reported for all populations of wild pigs^61^ and therefore it may depend on the intensity of persecution. Alternatively, nocturnal activity patterns might also be influenced by seasons and weather conditions^22,23,61^.

Interestingly, we found that even as the number of wild pig visits to bait sites declined as the population decreased, the time that wild pigs spent at bait sites did not appear to be affected, and remained high. This could be related to these animals relying on anthropogenic food sources during periods of food stress^17,19^. However, this result also indicated that wild pigs did not become conditioned to avoid using the bait sites, which has been suggested in other studies^22,24^. This is good news for elimination programs that use bait visitation as a strategy to detect wild pigs. Further, employing bait sites is a highly important method for finding the few remaining wild pigs, validating management actions and progress, or adopting different management strategies^31,32^.

Unsurprisingly, removals of wild pigs were also highest when more effort was put into activities associated with their removal, such as baiting, trapping, and shooting. However, the costs (and effort) increased exponentially to remove the last 1% of the wild pigs on the landscape, a relationship that has been similarly demonstrated in previous studies^62,63^. For example, the total cost of removing the three wild pigs that remained in Fulton County after April 2014, was $12,673. Strong community support and resources are needed during the final phases of an elimination program because locating and removing the last remaining wild pig takes excessive effort^34,64,65^. Dedication and support for removing these few remaining individuals is extremely important because just a small number of wild pigs can quickly become established as a prolific population^14,15,27^ and all progress sacrificed.

A primary challenge associated with this observational study was that wild pig removal was the primary goal, and estimating factors that optimized removal success were considered post-hoc. Thus, a rigorous study design to optimize precision of modeling efforts detailing other habitat predictors potentially significant at explaining bait site use by wild pigs was not employed. Also, we were unable to include demographic data in our analysis of monthly removals, which may have provided some insight into maximizing population reduction. Similarly, we were unable to examine how breeding seasons for wild pigs influenced population reduction for this population, because these seasons could not be defined since the population itself did not exist for long. Breeding seasons may vary by region and year for wild pigs^14,15,66^, and is likely important for timing of control efforts (e.g., increase effort just prior to farrowing). Future directions to further this work could include evaluating removal programs in other areas of the U. S. and with different primary removal strategies.

## Conclusions

Our study identified factors that may help increase efficiency of elimination programs for newly established populations of wild pigs. The timing of baiting activities can be strategically planned to maximize efficacy of removal efforts. Especially in more northern regions, increased effort in removing wild pigs should be focused during colder periods to maximize efficiency. Although wild pigs appear to be expanding in Canada^21,67,68^ and other northerly locations in North America^15^ which do experience extreme periods of cold temperatures, eradication campaigns may benefit from operating during prolonged periods of cold temperatures. Wild pigs did not become conditioned to avoid using bait sites, thus baiting was a valuable tool for aiding removal even as population densities become low. Elimination programs should be prepared for large expenditures and increased effort during the final stages to locate and remove the last remaining wild pigs. These results should aid in removal efficiency of future elimination programs where removing a small population of wild pigs is needed to prevent population expansion and damage to agriculture and the environment.
